# Syncytiotrophoblast derived extracellular vesicles transfer functional placental miRNAs to primary human endothelial cells

**DOI:** 10.1038/s41598-017-04468-0

**Published:** 2017-07-04

**Authors:** Tina Cronqvist, Dionne Tannetta, Matthias Mörgelin, Mattias Belting, Ian Sargent, Mary Familari, Stefan R. Hansson

**Affiliations:** 1 Lund University, Department of Clinical Sciences in Lund, Obstetrics and Gynecology, 22185 Lund, Sweden; 2 University of Reading, Department of Food and Nutritional Sciences, Whiteknights, Reading UK; 3 Lund University, Department of Clinical Sciences in Lund, Division of Infection Medicine, 22185 Lund, Sweden; 4 Lund University, Department of Clinical Sciences, Lund, Oncology and Pathology, 22185 Lund, Sweden; 5 Nuffield Department of Obstetrics and Gynaecology, John Radcliffe Hospital University of Oxford, OX3 9DU Oxford, UK; 6 School of Biosciences, University of Melbourne, Parkville, VIC 3010 Australia

## Abstract

During the pregnancy associated syndrome preeclampsia (PE), there is increased release of placental syncytiotrophoblast extracellular vesicles (STBEVs) and free foetal haemoglobin (HbF) into the maternal circulation. In the present study we investigated the uptake of normal and PE STBEVs by primary human coronary artery endothelial cells (HCAEC) and the effects of free HbF on this uptake. Our results show internalization of STBEVs into primary HCAEC, and transfer of placenta specific miRNAs from STBEVs into the endoplasmic reticulum and mitochondria of these recipient cells. Further, the transferred miRNAs were functional, causing a down regulation of specific target genes, including the PE associated gene fms related tyrosine kinase 1 (FLT1). When co-treating normal STBEVs with HbF, the miRNA deposition is altered from the mitochondria to the ER and the cell membrane becomes ruffled, as was also seen with PE STBEVs. These findings suggest that STBEVs may cause endothelial damage and contribute to the endothelial dysfunction typical for PE. The miRNA mediated effects on gene expression may contribute to the oxidative and endoplasmic reticulum stress described in PE, as well as endothelial reprogramming that may underlay the increased risk of cardiovascular disease reported for women with PE later in life.

## Introduction

Preeclampsia (PE) is a complex and severe pregnancy associated disorder and is diagnosed on the presence of newly developed hypertension and proteinuria from 20 weeks of gestation^1^. It is generally agreed that PE develops in two stages. During the first stage, the placenta is inadequately implanted which causes reduced placental perfusion, oxidative stress, increased placental apoptosis and excessive shedding of trophoblast debris. In the second stage, these components together with the anti-angiogenic factor, soluble receptor vascular endothelial growth factor (s-Flt), are released into the maternal circulation, where they cause systemic inflammation, endothelial dysfunction and organ failure^2, 3^. These factors, although not unique for PE, are present in excessive amounts during this disorder^4, 5^. Risk factors for PE include diabetes, obesity, previous PE pregnancies and chronic hypertension amongst others^6^.

Several placental factors have been suggested to link the first and second stage of PE. Studies from our research group suggest free foetal haemoglobin (HbF) may be an important factor in this transition^7^ as increased synthesis and accumulation of free HbF has been shown in PE placentas^8^. Further, leakage of HbF, from the damaged placenta, into the maternal circulation has been demonstrated both *ex vivo* ^9^ and in different cohort studies^10, 11^. The negative effects of free HbF and its metabolite heme on placental function have been confirmed in several animal studies^12–14^. By perfusing human normal placentae *ex vivo* with free Hb, an increased perfusion pressure and induction of PE-like pathological changes, as well as increased placental cell blebbing and formation of apoptotic vesicles, was shown^9, 15^. Based on these studies, we hypothesized that free HbF may be responsible for inducing the increased shedding of syncytiotrophoblast extracellular vesicles (STBEVs) evident in PE^16, 17^.

Extracellular vesicles (EVs) are membrane vesicles released by all cells studied to date, and divided into exosomes and microvesicles based on size and site of formation in the cell. Exosomes are released by exocytosis from multivesicular bodies and have a size ranging between approximately 30–100 nm. Microvesicles are shed directly from the plasma membrane and range from 100nm-1µm in diameter. The placenta also releases syncytial nuclear aggregates (20–500 µm) as well as apoptotic bodies (1–4 µm)^18, 19^. Placental EVs are often referred to as STBEVs due to their syncytiotrophoblast cell of origin, and they are believed to play an important role both in normal and dysfunctional pregnancies. It has been suggested that STBEVs adapt the maternal immune system to the on-going normal pregnancy^20, 21^. In PE, the plasma level of STBEVs is increased and PE STBEVs show different characteristics compared to normal STBEVs. For example, PE STBEVs show increased expression of Tissue Factor (TF)^18, 22^, which may trigger the immune system to be more active and/or damaging^16, 17^. It is also known that PE STBEVs are larger in size compared to normal STBEVs^23^. The STBEVs are believed to be one factor that causes the endothelial dysfunction seen in PE^24^.

STBEVs carry^15, 25^ and transfer miRNAs to recipient cells^26–28^. miRNAs are short non-coding RNAs, which affect gene expression either by degrading mRNA, or by inhibiting mRNA translation^29^. The chromosome 19 miRNA cluster (C19MC) has been shown to be almost exclusively expressed in the placenta^30–32^ and C19MC miRNAs are found in placenta released STBEVs^25, 26, 28, 33^. C19MC miRNAs have been characterized in the maternal circulation and their expression profile is altered in PE^34, 35^. We have reported that Hb perfusion of human placentas alter the miRNA content of released STBEVs from normal placentas^15^.

Several studies have shown increased arterial stiffness – an indicator of endothelial dysfunction, in women with PE – correlating with PE severity^24^. Women with PE have an increased risk of cardiovascular disease later in life, possibly due to PE-induced endothelial and vascular dysfunction^36, 37^. We hypothesize that the increased levels of STBEVs together with free HbF play an important role in endothelial dysfunction, by inducing oxidative stress and membrane damage. We also hypothesize that STBEVs transfer potentially functional miRNAs, to endothelial cells to alter gene expression, leading to cellular alterations that cause long term changes in the endothelium. In the present study, we investigated the uptake of STBEVs, isolated from perfused placentas, from normal and PE pregnancies, by primary human coronary artery endothelial cells (HCAECs). To mimic the double hit seen in PE, with altered STBEVs as well as increased HbF, the role of free HbF on the STBEV uptake was specifically studied. We also investigated the transfer of placental miRNAs and their effects on endothelial cell target gene expression.

## Results

### Characterisation of STBEVs

#### Nanoparticle Tracking Analysis

**Figure 1:**
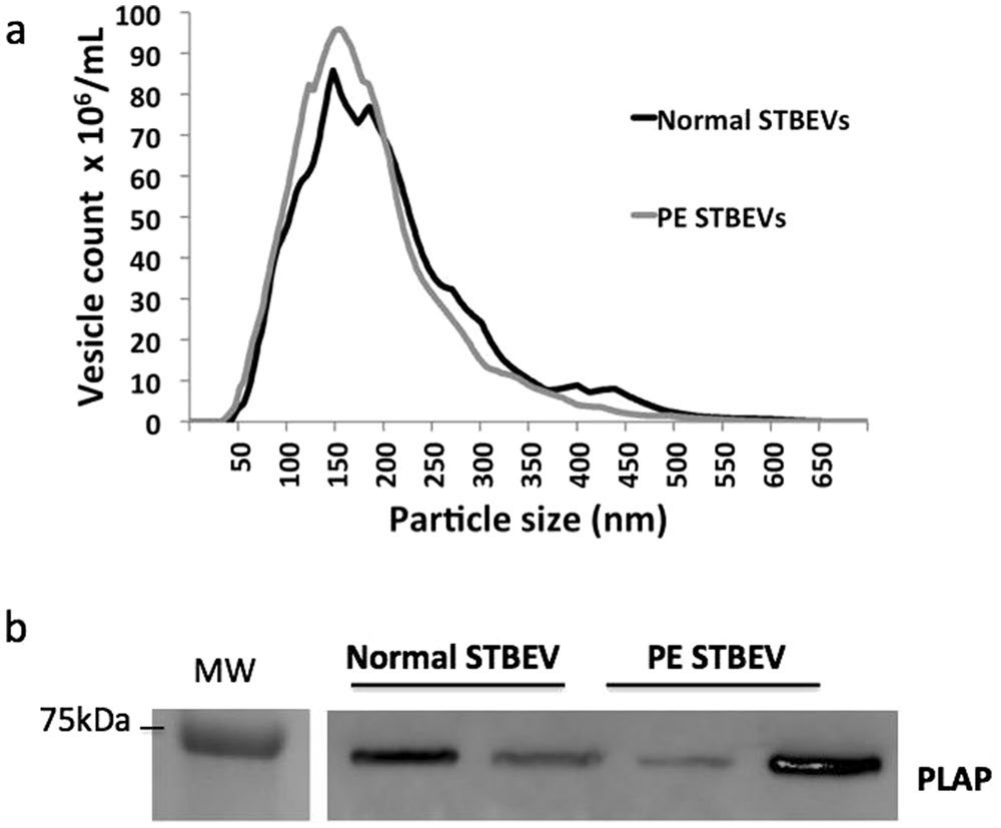
Characterisation of STBEVs using NTA and western blotting. Using Nanoparticle Tracking Analysis (NTA), vesicle size distribution and concentration was determined. The NTA showed STBEV vesicle sizes ranging between 50–500 nm (**a**) for both normal and PE STBEVs. Both normal and PE STBEV samples were PLAP positive by western blotting (**b**), shown by using the primary antibody NDOG2 against the STBEV surface marker PLAP (60 kDa). Each lane represents one individual sample. Blots have been cropped and inverted, see Supplementary Information S5 for full-length blots. Please note that the molecular marker shown in this figure is from a different blot run simultaneously, also shown in Supplementary Information S5.

To determine vesicle count and size distributions of normal and PE STBEVs, we used the Nanoparticle Tracking Analysis (NTA) methodology. Mean vesicle sizes were 206 ± 20 nm and 195 ± 27 nm for normal and PE STBEVs respectively (Fig. 1a). No significant differences were observed in terms of vesicle count or size ranges between groups.

#### Western Blot

Western blot analysis showed that both normal and PE STBEV samples were PLAP positive, consistent with their placental syncytiotrophoblast origin (Fig. 1b).

### STBEV uptake in primary human coronary artery endothelial cells

#### Time course assessment of STBEV uptake

**Figure 2:**
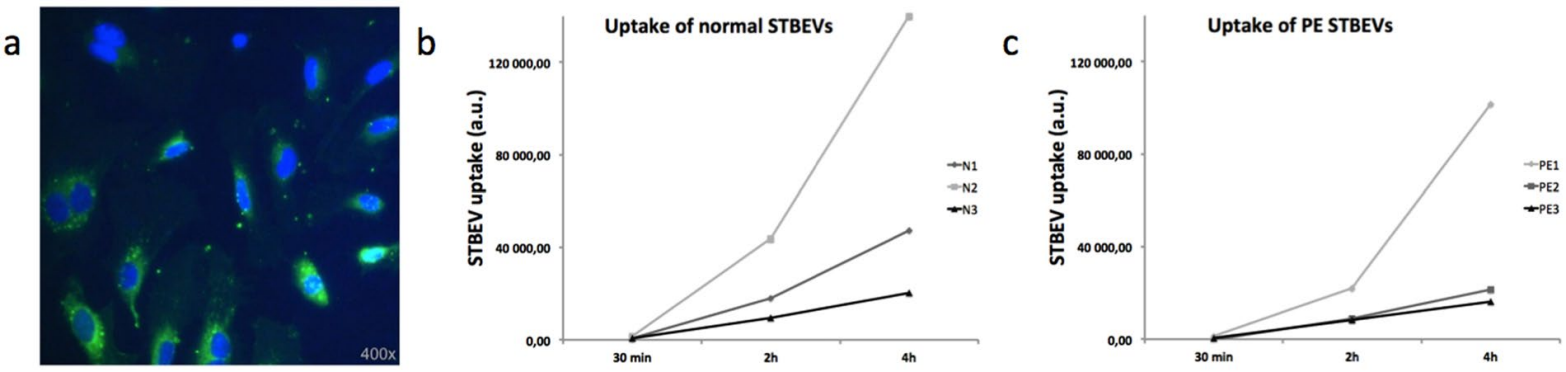
Time course assessment of STBEV uptake. HCAEC uptake of PKH-labelled STBEVs was visualised using fluorescent microscopy (**a**). A representative image of HCAECs following 6 hours of incubation with PKH-labelled STBEVs. The cell nuclei are labelled blue and PKH-labelled STBEVs are seen as green. Time-dependent uptake of normal (b, N1-N3) and PE (c, PE1-PE3) STBEVs was quantified using flow cytometry. Although variable between samples, all samples showed a time-dependent uptake. The horizontal axis shows mean fluorescent values; a.u., arbitrary units.

The uptake of PKH-labelled STBEVs in primary HCAECs was visualized by fluorescence microscopy (Fig. 2a) and quantified over time using flow cytometry (Fig. 2b). There was no obvious uptake after 15 minutes incubation, but uptake was detected after 30 minutes and increased throughout the 24 hours time course. At 24 hours, all visualised cells appeared positive for PKH-stained STBEVs. There were no apparent differences in uptake pattern between normal and PE derived STBEVs, with respect to time-course or number of PKH-STBEV positive cells.

#### Confocal microscopy

**Figure 3:**
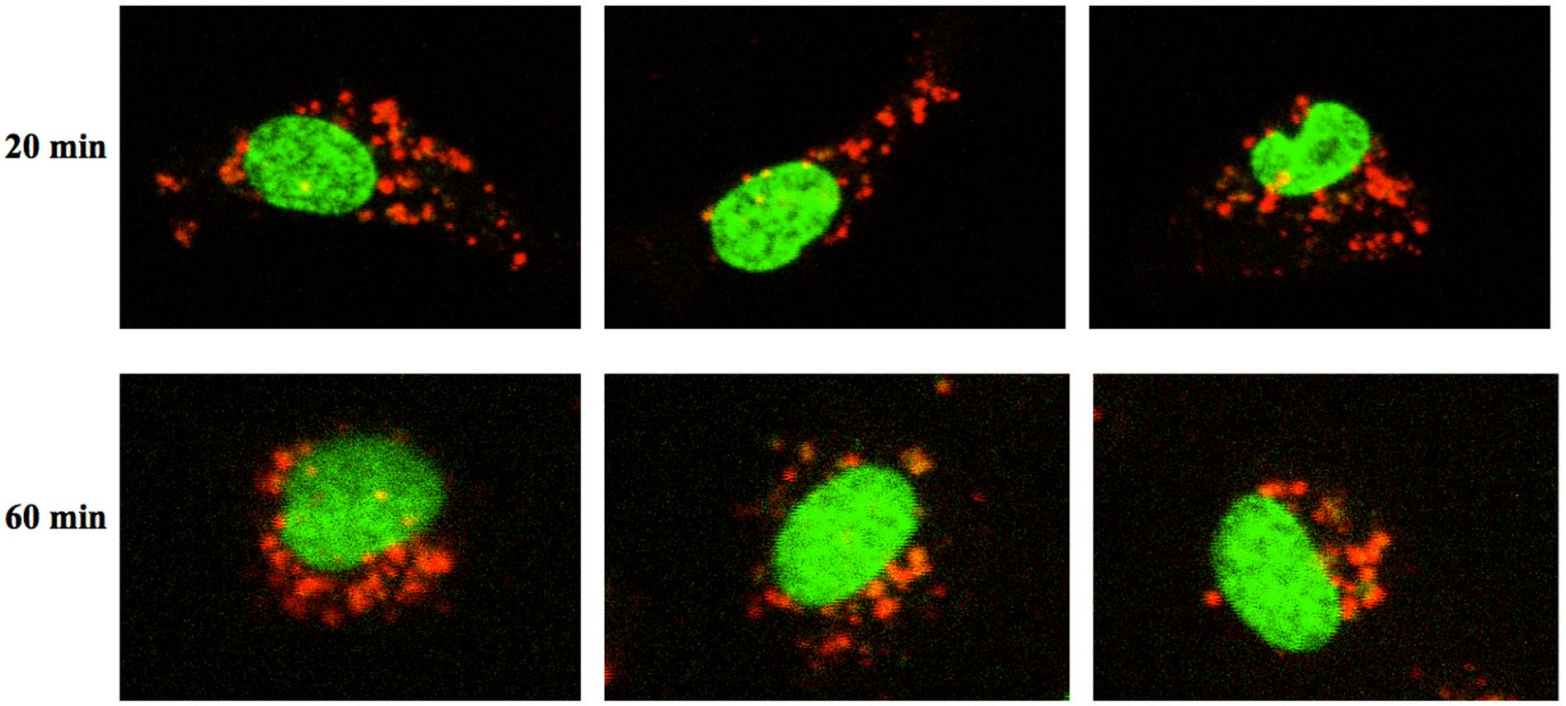
Live confocal microscopy visualizing STBEV uptake and intracellular transport. Uptake of PKH-labelled STBEVs into HCAECs was followed by live confocal microscopy. Three representative cells are shown after 20 and 60 min of incubation. After 20 min the STBEVs are clearly internalised and widely distributed in the cell cytoplasm. After 60 min the STBEVs appear to be closer to the cell nuclei. Cell nuclei are pseudocolored in green, and PKH-labelled STBEVs in red. The figures are from live imaging, explaining the lower intensity at 60 minutes due to bleaching.

PKH-labelled STBEV internalization was confirmed by confocal microscopy, clearly showing that the STBEVs were not located on the cell surface (Fig. 3). The dynamics of internalized STBEVs could be further visualized by real-time confocal imaging (Supplementary information S1: video). Confocal imaging was more sensitive than flow cytometry analysis, and this analysis clearly demonstrates internalised STBEVs at 20 min. At 60 min of internalisation STBEVs were not as widely distributed in the cell cytoplasm, but appeared closer to the cell nucleus (Fig. 3).

### STBEV transfer of miRNAs to primary human coronary artery endothelial cells

#### Micro-RNA analysis

Total RNA was extracted from HCAEC cultures treated with normal or PE STBEVs. The miRNAs belonging to the C19 miRNA cluster (C19MC) and the 371–373 cluster, as well as four commonly expressed miRNAs (mir-210; mir-222; mir-16; mir-141*) were analysed using a custom TaqMan® miRNA array card from Applied Biosystems (The full list of miRNA sequences are listed in Supplementary information S2). The miRNAs showing expression levels at the cut-off value Ct <35 in all triplicates for one or more samples were included for further analysis. Out of the 52 C19MC miRNAs examined, 12 miRNAs fulfilled these criterion (marked with # in S2, Ct values presented in Supplementary information Table S3). Of special interest were the three C19MC miRNAs mir-517c, mir-517a and mir-519a, which were expressed in a majority of the samples but not in the controls, i.e. untreated cells. None of the 371–373-cluster miRNAs, fulfilled the cut-off criteria. Mir-210, mir-222 and mir-16, were expressed in all samples including the controls. Mir-141* was not detected in any samples. In the array analysis, there was significantly lower expression of mir-210 in the PE STBEV treated cells compared to cells treated with STBEVs from normal pregnancies (p = 0.022, Table S3). The mir-222 showed significantly higher expression in the cells treated with normal STBEVs compared to the untreated control cells (p = 0.031, Table S3). These differences however could not be confirmed using real time quantitative PCR (RTqPCR). There was no significant difference between normal and PE STBEV treatments (Table S3). The endogenous control U6 snRNA was stably expressed throughout all samples.

**Figure 4:**
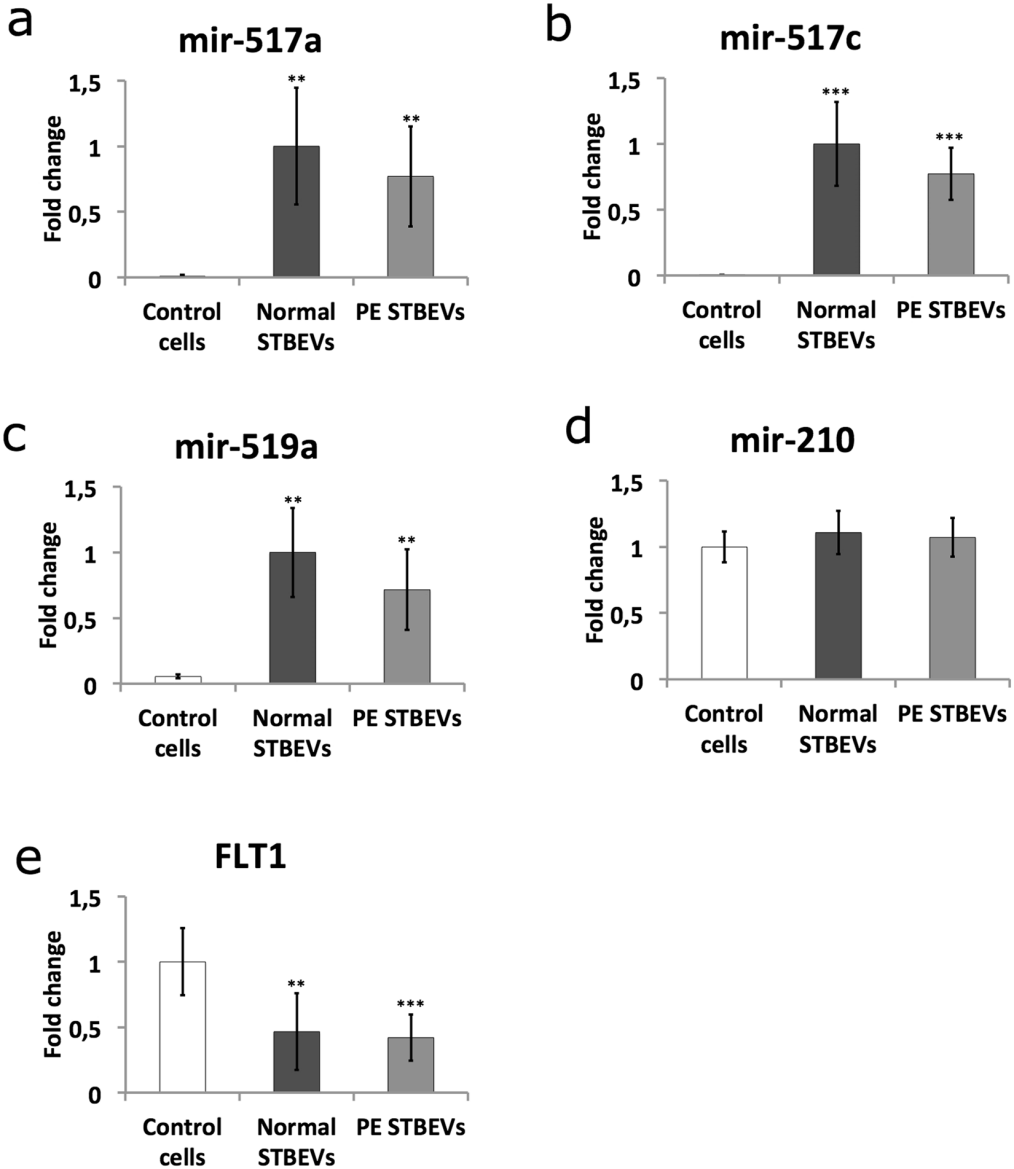
Micro-RNA and target gene expression in primary human coronary artery endothelial cells following STBEV treatment. The three placental C19MC miRNAs, mir-517a (**a**), mir-517c (**b**) and mir-519a (**c**), were analysed using RTqPCR. None of the three C19MC miRNAs were present in un-treated control cells. After treatment with normal (n = 5) or PE (n = 5) STBEVs, the levels of all three miRNAs increased significantly compared to control cells. No significant difference was seen in miRNA expression when comparing normal and PE STBEV treatments for any of the three C19MC miRNAs. The mir-210 (**d**) was present in controls cells and unaffected by STBEV treatment. The miRNA expression is expressed as fold change (±SD), which were calculated by normalizing against cells treated with normal STBEVs. Expression level of the predicted target gene FLT1 (**e**) was down regulated after normal (n = 5) and PE (n = 5) STBEV treatment. FLT1 is shown here as representative of the four genes; FLT1, TGFBR2, PDGFD and PDCD1LG2, all affected similarly by STBEV treatment. See also Fig. 5. The expression of predicted target genes was calculated as fold change (±SD) by normalizing against control samples, i.e. untreated control cells. Differences between control and normal STBEV treatments, as well as between control and PE STBEV groups, were analysed using Mann-Whitney *U*-test. *p < 0.05, **p < 0.01, ***p < 0.001.

Based on the array results, four miRNAs (mir-517a, mir-517c, mir-519a and mir-210) were selected for further analysis using RTqPCR. Control cells, i.e. not treated with STBEV, did not express these three C19MC miRNAs. The analysis confirmed the presence of the three C19MC miRNAs in cells treated with either normal or PE STBEVs (Fig. 4a). After treatment with normal STBEVs, the levels of mir-517a, mir-517c and mir-519a increased significantly (p = 0.00164, p = 0.0002 and p = 0.00164 respectively). Treatment with PE STBEVs also caused a significant increase (p = 0.00124, p = 0.00012 and p = 0.00124 respectively). The mir-210 expression levels remained stable in the cells regardless of treatment group (Fig. 4b), most likely reflecting the cells endogenous mir-210 expression. This also suggests that the STBEVs contain very low levels of mir-210, consistent with previous studies showing that trophoblast cells release vesicles mainly containing C19MC miRNAs^25^.

#### Micro-RNA target genes

**Figure 5:**
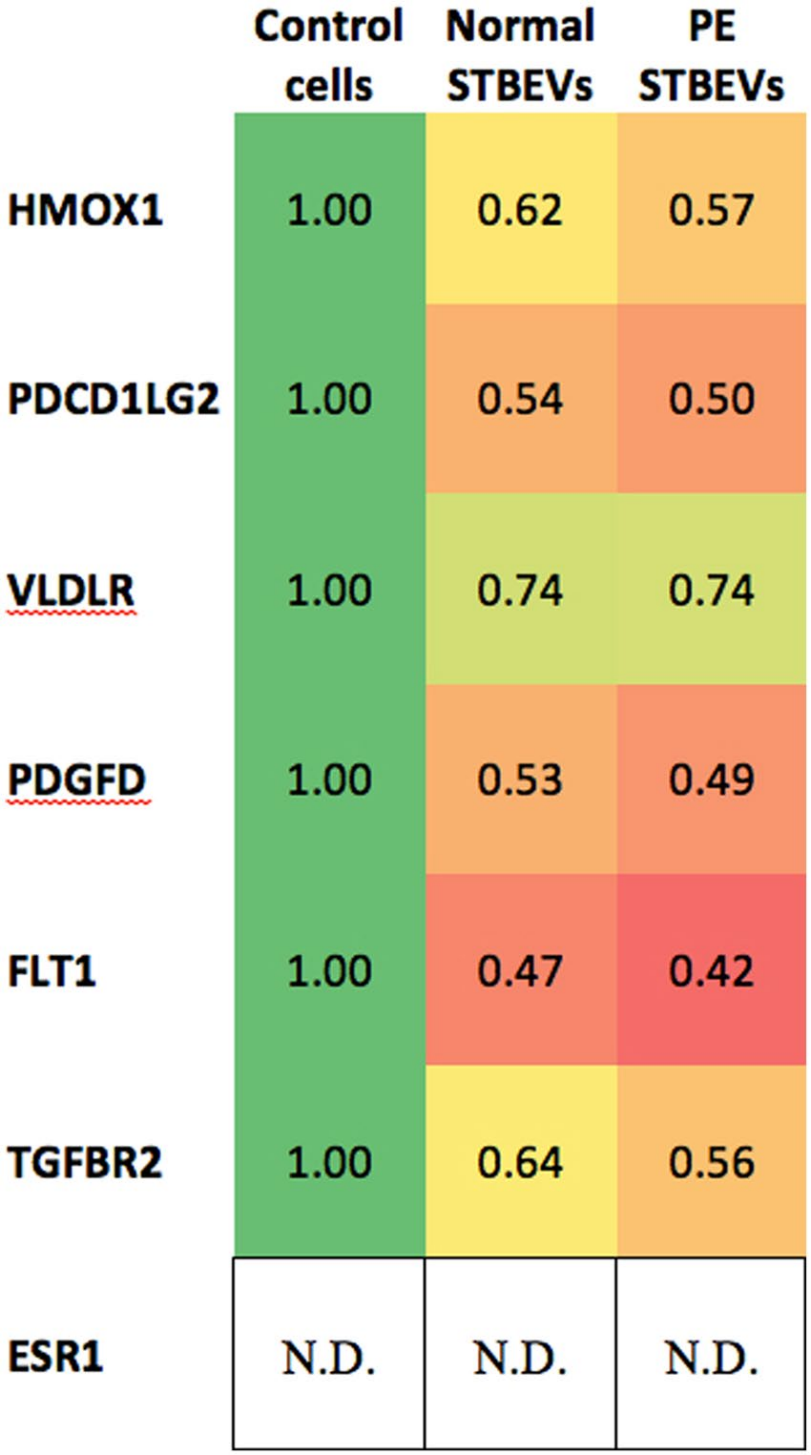
Target gene fold changes in HCAECs, after treatment with normal or PE STBEVs compared to controls. Heat-map showing fold change for each gene relative to control cells. A deviation from 1.0 indicates down-regulation of gene expression. N.D (not detected) in HCAEC cells.

The strategy for predicting miRNA targets is detailed in the Methods section. Briefly, by using three different prediction algorithms, as well as the TarBase database for experimentally validated miRNA targets, we identified putative target genes for the miRNAs mir-517a, mir-517c and mir-519a (Fig. 5). The following genes were analysed by RTqPCR; fms related tyrosine kinase (FLT1), transforming growth factor beta receptor 2 (TGFBR2), platelet derived growth factor D (PDGFD), very low density lipoprotein receptor (VLDLR), estrogen receptor 1 (ESR1) and programmed cell death 1 ligand 2 (PDCD1LG2). As an indicator of cell stress, heme oxygenase 1 (HMOX1) was analysed. The VLDLR mRNA expression remained unchanged after both normal (p > 0.05) and PE (p = 0.039, fold change <1.5) STBEV treatment. The ESR1 mRNA expression was not detectable in our samples. Normal STBEV treatment led to significant down regulation of the genes FLT1 (p = 0.0014), TGFBR2 (p = 0.0074), PDGFD (p = 0.0074), PDCD1LG2 (p = 0.0033) and HMOX1 (p = 0.0096), In the same manner, PE STBEV treatment lead to significant down regulation of FLT1 (p = 0.0005), TGFBR2 (p = 0.0021), PDGFD (p = 0.0012), PDCD1LG2 (p = 0.0012) and HMOX1 (p = 0.0071). There was no statistically significant difference comparing normal and PE STBEV treatment (Figs 4c and 5).

### Transmission Electron Microscopy

#### STBEV characterisation

The placental marker PLAP was visualized on vesicles of all sizes using Transmission Electron Microscopy (TEM) (Supplementary Figure S4). The estimated size range was 10–400 nm in both normal and PE (Supplementary Figure S4a) samples, which was in good agreement with the NTA data in Fig. 1a. By using a gold-labelled miRNA primer (hsa-mir-517c), mir-517c could be visualized inside both PLAP positive and PLAP negative STBEVs, the PE STBEVs shown in Supplementary Figure S4b. Furthermore, mir-517c was also localized outside of the vesicles, in what appeared to be protein complexes. HbF was visualized with HbF specific antibodies inside both PLAP positive and PLAP negative PE STBEVs of all sizes (Supplementary Figure S4c). Normal STBEVs did not contain HbF (Supplementary Figure S4d).

#### Cell analysis

**Figure 6:**
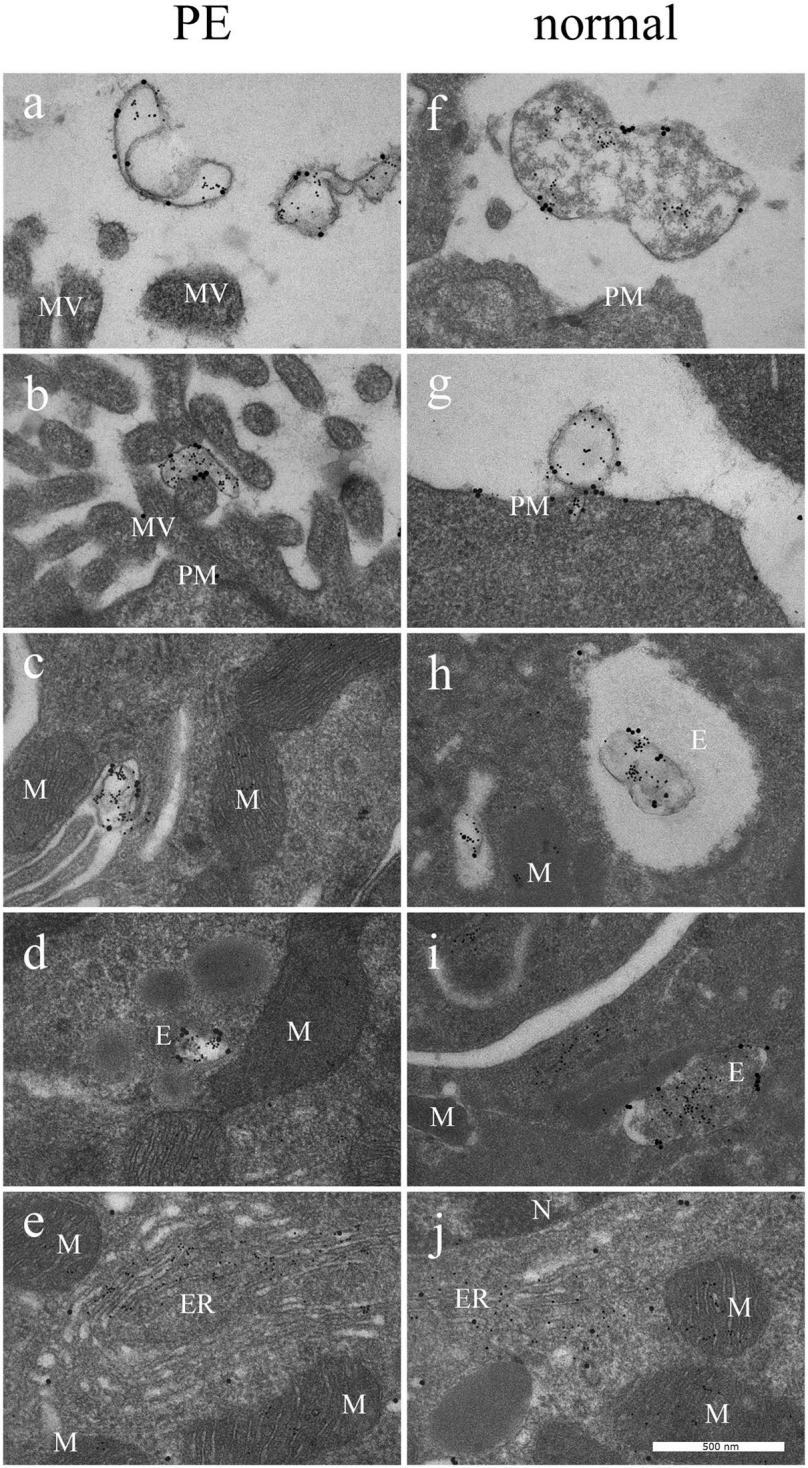
STBEV uptake and miRNA transfer visualised by transmission electron microscopy. By using TEM, PE STBEVs (**a–e**) and normal STBEVs (**f–j**) were visualized in HCAECs. In panel (a), PE STBEVs approach the HCAEC. In (**b**) and (**c**), PE STBEV appears to be binding to the ruffled plasma membrane. The STBEV is seen inside the cells in what appears to be an endosome (**d**), in close proximity to the mitochondria, endoplasmic reticulum and cell nucleus. The mir-517c from PE STBEVs appears to be in larger number in the ER (**e**) compared to the mitochondria. PLAP appears to stay in the endosomes and is possibly recycled to the cell membrane. The normal STBEVs, which also carry mir-517c are found outside the cells (**f–g**) and can be found in endosomes (**h,j**). In contrast to PE STBEVs, mir-517c from normal STBEVs are localized in a higher degree to mitochondria and in lower quantity to the ER (**j**). The STBEV and miRNA intracellular distribution are also described in Table 1. Abbreviations; E endosome, ER endoplasmic reticulum, MV microvilli, M mitochondria, N nucleus, PM plasma membrane. PLAP labelled with 20 nm and mir-517c labelled with 5 nm colloidal gold.

**Figure 7:**
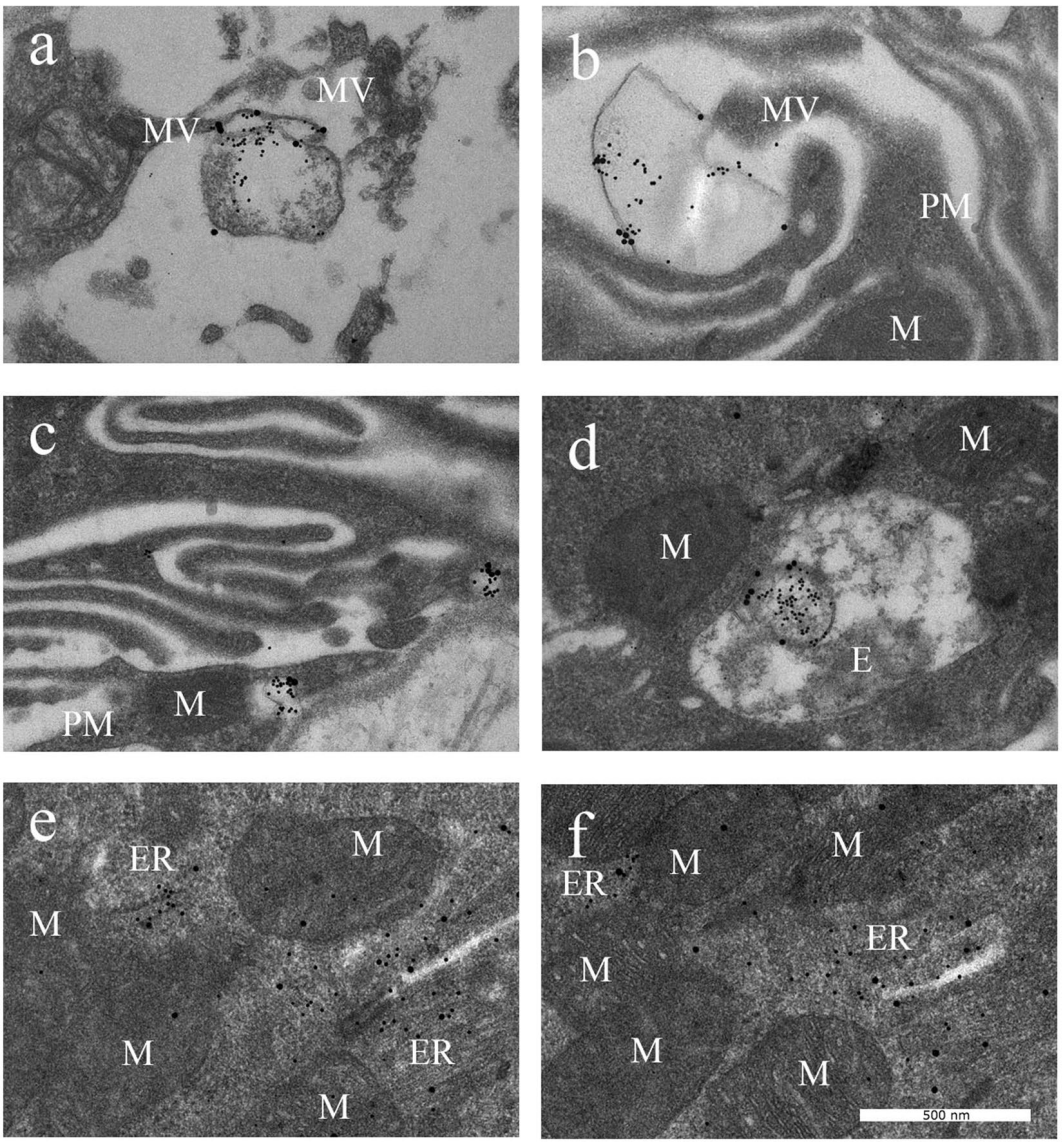
STBEV uptake and effect of free HbF visualised by transmission electron microscopy. The normal STBEVs, co-treated with HbF, appear to bind the plasma membrane (**a**), which is ruffled (**b,c**) as was also seen in cells treated with PE STBEVs (see Fig. 6b). The STBEVs were internalised into endosomes (**d**) and transported to intracellular compartments (**e,f**). The mir-517c is localised predominantly into the ER than in the mitochondria (**e,f**), also seen in cells treated with PE STBEVs (see Fig. 6e). Abbreviations; E endosome, ER endoplasmic reticulum, MV microvilli, M mitochondria, N nucleus, PM plasma membrane. PLAP labelled with 20 nm and mir-517c labelled with 5 nm colloidal gold.

Uptake of STBEVs into HCAECs was visualized using TEM. The placental marker PLAP was located on vesicles outside the cells, bound to the cell surface, as well as inside the endothelial cells in different compartments (Fig. 6). The PLAP marker was also found to be recycled to the cell membrane (Fig. 6g). Both normal and PE STBEVs were positive for PLAP and mir-517c. The mir-517c remained co-localized with PLAP positive vesicles until the STBEVs reached the endosomes. Hereafter, the STBEVs appeared to be degraded, whereby PLAP was possibly recycled to the cell surface, and mir-517c re-located to either the endoplasmic reticulum (ER) or the mitochondria. The normal STBEVs appeared to deposit their miRNA content in higher quantity to the mitochondria (Fig. 6j, Table 1). In contrast, the PE STBEVs were deposited in a higher degree to the ER (Fig. 6e, Table 1). With TEM, the intracellular distribution could be followed, although it is worth mentioning that these results are primarily descriptive and not established statistically. Furthermore, treatment with PE STBEVs caused extensive damage to the endothelial cell membrane inducing membrane ruffling (Fig. 6b), which was not seen after treatment with normal STBEVs (Fig. 6g). The primary endothelial cells were also exposed to a combination of HbF and normal STBEVs (Fig. 6), to evaluate the potential role of the HbF found in the PE STBEVs. A severe membrane ruffling occurred (Fig. 7b and c), similar to that seen in cells treated with PE STBEVs. The normal STBEVs were taken up into endosomes (Fig. 7d), but the co-treatment with HbF resulted in miRNA deposition shifting towards the ER (Fig. 7e and f), as was seen for PE STBEVs.

**Table 1:** Semi-quantification of miRNA gold label per square micrometre in Transmission Electron Microscopy data.

	Normal STBEVs (n = 100)	PE STBEVs (n = 100)	Normal STBEVs+HbF (n = 100)
Plasma membrane	43 ± 10	69 ± 15	81 ± 18
Cytoplasm	39 ± 11	34 ± 12	36 ± 11
Endosomes	54 ± 9	66 ± 13	71 ± 12
Mitochondria	42 ± 6	28 ± 8	31 ± 10
ER	21 ± 4	39 ± 8	41 ± 9

## Discussion

The present study provides evidence that STBEVs are internalized into HCAECs, and that STBEV cargo, placenta specific miRNAs, is transferred into recipient cells. To our knowledge, no previous uptake studies have been performed on HCAECs using STBEVs from perfused placentas. Previous studies have visualized the uptake of mechanically derived placental or cell-culture derived syncytiotrophoblast vesicles into cells such as vascular smooth muscle cells^38^, BeWo cells or primary villous cytotrophoblasts^39^.

The results show that STBEVs are internalized with increased uptake after longer incubation time, lending support to the notion that uptake of STBEVs into HCAECs is active and involves a specific uptake mechanism. The evidence for STBEV internalization was obtained both indirectly by flow cytometry, as well as directly by confocal microscopy live imaging and TEM. The flow cytometry analysis was also used to quantify the time-dependent uptake observed *in vitro*. In our hands, the STBEV uptake was significant after 15–30 minutes, with increasing uptake throughout the 24-hour time course. Prior to internalization, the STBEVs appeared to bind to the cell surface. Using TEM, we showed that the STBEVs were transported to endosomes, where it is possible that the vesicles were degraded and re-cycled to the cell-surface, as appears to be the case for PLAP labelled vesicles. The uptake into endosomes suggests that the STBEVs are, at least in part, taken up by endocytosis, but the presence of PLAP on the plasma membrane, could also suggest a partial uptake through membrane fusion. The exact uptake mechanisms and whether these differ between EV subgroups of exosomes and microvesicles, remains to be elucidated^18^.

The miRNA content (specifically mir-517c) was, after vesicle degradation, transported primarily to the rough endoplasmic reticulum (ER) or the mitochondria. Localization of miRNAs to the ER suggests that the miRNAs are actively involved in regulating gene expression at the translational level. Data on target gene expression further supports the idea that STBEV miRNAs are functional after uptake, since several C19MC miRNA predicted or validated target genes were down regulated. Previous studies have also shown vesicles affecting target gene expression in recipient cells. For example, human umbilical vein endothelial cells (HUVECs) were shown to take up plasma-EVs derived from pregnant women, and several studies have reported that STBEV had effects on both gene expression and cellular functions^40–42^. In addition there were different effects depending on whether the EVs were derived from normal or PE patients^43^. In the present study we saw no difference in target gene down-regulation when comparing normal and PE STBEVs. The miRNA expression data suggests that normal and PE STBEVs contain the same amount of C19MC miRNAs, and the cells were treated with equivalent concentrations of STBEVs, which could explain why the effect on gene expression is similar. Another possible explanation is that there could be long-term effects after several months’ treatment, as is the case in pregnant women. The ruffled plasma membrane caused by PE STBEVs may lead to cellular dysfunction over time.

Interestingly, by semi-quantifying the gold labelled STBEVs with TEM, we saw that normal STBEV miRNAs were deposited at a higher degree in the mitochondria, while PE STBEV miRNAs primarily were deposited to the ER. We speculate that this could be due to different molecules or tags on the EV surface, directing the intra-cellular transport of vesicles to different subcellular compartments. In previous studies, HbF has been shown to affect both mitochondria and ER^14^ and PE placentas are subject to high levels of both oxidative and ER stress^44^.

This led us to the hypothesis that the HbF inside PE STBEVs cause oxidative stress and damage to the mitochondria or ER, thereby re-directing the STBEVs and their miRNA cargo. The extensive membrane ruffling, seen after treatment with PE STBEVs as well as with normal STBEVs co-treated with free HbF, suggests destruction of the cytoskeleton, which also disrupts intracellular pathways. A functioning cytoskeleton is required for EV uptake through endocytosis^45^. The re-direction of STBEV cargo and shifted balance of STBEV miRNA deposition from the mitochondria to the ER could increase the ER stress further. This could possibly affect the translation of various proteins important for normal pregnancy adaptation. As shown here, even normal STBEVs transfer unique information in the form of placenta specific miRNAs. And this may be important for placenta-maternal communication.

Our TEM studies showed that PE STBEVs carried HbF. Since free HbF is a toxic molecule^3^ this could have damaging effects on endothelial cell structure and function, and may play an important role in causing the endothelial dysfunction characteristic of PE^4^. By co-treating normal STBEVs with HbF, similar cell membrane ruffling was seen, as with PE STBEVs alone, lending support to the notion that HbF is at least one of the harmful differences between normal and PE STBEVs. Various conditions have previously been shown to affect different EV uptake pathways. For example, blocking specific receptors with heparin or Annexin-V, inhibits endocytic uptake via protein-protein interactions. Further, acidic conditions increase uptake through membrane fusion, whereas low temperature (4 °C) inhibits the energy-requiring uptake through endocytosis^45^. We speculate that HbF may enhance or inhibit certain uptake pathways or intra-cellular transport mechanisms, for example by disrupting the cytoskeleton or inducing oxidative stress.

Regarding STBEV characterisation, the NTA data showed no difference in vesicle count or size distribution, between normal and PE samples. It is well documented that STBEVs increase significantly in the plasma of women with PE^22^. However, at present it is not possible to normalize the surface area of a perfused placenta, and generally only one cotyledon is perfused, and these vary in size even within a single placenta. Therefore the number of STBEV released per perfused cotyledon is variable. Further, the vesicle size can be affected by freezing^46^, which may explain the differences between the groups. We therefore draw no conclusions in this study regarding the STBEV count in plasma from pregnant women.

In conclusion, the results show STBEV uptake and internalization by HCAECs, and transfer of placenta specific miRNAs, which in turn affect predicted and validated target gene expression. Normal and PE STBEVs deposited their miRNA content in different cellular compartments and co-treatment with free HbF and normal STBEVs, re-directed the miRNA deposition to a pattern similar to that seen in cells treated with PE STBEVs. Also, PE STBEVs caused extensive cell membrane damage, not seen with normal STBEVs. In PE, there is a higher plasma concentration of both STBEVs and free HbF, which may lead to re-programming of endothelial cells that may cause detrimental cellular functions such as arterial stiffness^24^. This may explain the long-term cardiovascular consequences seen in women who have suffered PE during pregnancy^37^.

## Methods

### Ethics statement

The perfusion studies were approved by the Oxfordshire Research Ethics Committee C at Oxford University, and informed written consent was obtained from all participants. All experiments were performed in accordance with relevant guidelines and regulations.

### Placental perfusion and sample collection

Dual *ex-vivo* perfusion of isolated human placental cotyledons from normal (n = 5) and PE (n = 5) pregnancies was performed as previously described^47^. Briefly, the perfusion experiment consisted of one equilibration phase of 30 minutes, from which the maternal perfusate was discarded. After equilibration, the maternal circuit was closed and the placenta was perfused for 3 hours. Only perfusions with a foetal return rate of >80% were included. The perfusate was collected from the maternal side at the end of the 3 hours perfusion and used for isolation and analysis of STBEVs. The placental perfusion method was chosen for collecting normal and PE STBEVs in this study, due to the large vesicle yield that is available using this method, and the possibility to study STBEVs from syncytiotrophoblasts in PE placentas^48^.

### Isolation of STBEVs

The STBEV isolation was performed using sequential centrifugation steps. After perfusion, the maternal perfusate was centrifuged at 600 × g or 1500 × g to remove red blood cells, and the supernatant was frozen at −80 °C until further analysis. The supernatant was centrifuged for 30 minutes at 3,500 × g and 4 °C to remove cellular debris. The 3,500 × g supernatant was further ultra-centrifuged for 3 hours at 110,000 × g at 4 °C, and the pelleted material used for further study and referred to as the STBEVs. The STBEV protein concentrations were determined using a NanoDrop Spectrophotometer ND-1000 (NanoDrop Technologies, Wilmington, USA), or using the Pierce™ BCA Protein Assay (Thermo Scientific, Rockford, USA). The STBEVs were re-suspended in 1xPhosphate Buffered Saline (PBS), and stored at −80 °C.

### STBEV characterisation using Nanoparticle Tracking Analysis

For quantification and determination of size distribution, the STBEVs were analysed using NTA, with the NanoSight LM10 (Nanosight Ltd., Amesbury, UK) equipped with 635 nm laser. The STBEV preparations from normal (n = 5) and PE (n = 5) placentas, isolated by ultra centrifugation, were immediately diluted 1:1000 in 1xPBS and frozen at −80 °C until analysis. The diluted samples was introduced into the sample chamber at room temperature (22.7 ± 0.23 °C) using 1 mL syringes, and the chamber was washed between samples with PBS. For each sample, a 30-second video was recorded, using a camera gain of 400. The video was analysed using the NTA 2.3 software (NTA 2.3 build 0356 from Nanosight Ltd). Camera level was set to 14 and detection threshold set to 10. Automatic settings were used for all other parameters.

### STBEV characterisation using Western Blot

For identification of the STBEV surface marker placental alkaline phosphatase (PLAP), western blotting was carried out on STBEV lysates. Briefly, 10ug/well per sample of total protein from normal STBEVs (n = 2) and PE STBEVs (n = 2) were separated on 4–20% bis-tris gradient gel (Bio-Rad, Hercules, CA, USA), using molecular weight standard (Precision Plus All Blue, Bio-Rad). Separated proteins were transferred onto PVDF membranes, and nonspecific binding blocked with 5% Blotting-Grade Blocker (Nonfat Dry Milk, Bio-Rad) in PBS-Tween. The membranes were incubated with the primary antibody NDOG2 against PLAP (made in-house and provided by Prof Sargent) overnight at 4 °C. Membranes were washed prior to incubation with the secondary antibody Alexa Fluor 647 goat anti-mouse IgG (Life Technologies, Carlsbad, CA, USA), washed again and thereafter the bands were detected in a ChemiDoc XRS unit (Bio-Rad).

### Cell culture

Human coronary artery endothelial cells (HCAECs, Lonza Walkersville, Inc., MD, USA) were cultured in endothelial cell basal medium-2 supplemented with human epidermal growth factor (hEGF), hydrocortisone, human recombinant fibroblast growth factor-beta (hFGF-b), vascular endothelial growth factor (VEGF), insulin-like growth factor (R3-IGF-1), ascorbic acid, fetal bovine serum (FBS) and gentamicin/amphotericin-B (GA). The cells were cultured in a humidified incubator with a gas supply of air and 5% CO_2_ at 37 °C.

### STBEV uptake in primary human coronary artery endothelial cells

#### Fluorescent labelling of STBEVs

Using the PKH67 Fluorescent Cell Linker Kit (Sigma-Aldrich, St. Louis, MO, USA), the STBEV membranes were labelled with PKH67 dye according to manufacturer’s instructions. Briefly, the STBEV sample was mixed with diluent C and dye for 5–10 minutes, and the reaction stopped by adding FBS. For the initial time course assessment, using conventional fluorescence microscopy, the dyed STBEVs were recovered by centrifuging the solution for 30 minutes at 20,000 × g, the pellet washed once with 1xPBS and repeating the centrifugation step for an additional 30 minutes. The final pellet was resuspended in 1xPBS. For confocal microscopy and flow cytometry analysis, the dyed STBEVs were washed and recovered by centrifuging for 3 hours at 100,000 × g twice before resuspension in 1xPBS.

#### Time course assessment using fluorescence microscopy

The HCAECs were plated on gelatine (1%) coated cover slips and incubated overnight. The following day, the medium was changed and PKH-labelled STBEVs (20ug/mL) added to the cultured cells, or vehicle only for controls (no STBEV treatment). The experiment was ended at specific time points; 15 minutes, 30, 45, 60, 90 and 120 minutes, and 6 or 24 hours. The media was removed and 2 mL Hoechst 33342 (1 µg/mL, Life Technologies), nuclear stain diluted in 1xPBS, was added for 5 minutes. The cells were washed with 1xPBS before adding 2 mL 4% paraformaldehyde (PFA) (HistoLab Products AB, Gothenburg, Sweden) for 10 minutes. The cells were once again washed with 1xPBS, the cover slips removed, tapped dry and mounted on to a microscope slide using Fluoromount^TM^ Aqueous Mounting Medium (Sigma-Aldrich). The cells were visualized using a Zeiss Axiostar plus microscope at 100x and 400x magnification.

#### Time course assessment using flow cytometry

The HCAECs were plated out in 24 well plates overnight, and the following day, treated with PKH-labelled normal (n = 3) or PE (n = 3) STBEVs (5ug/mL) in serum free medium for 30 minutes, 2 hours or 4 hours. All samples were analysed in duplicates or triplicates. Cells were detached by trypsinization and washed and resuspended in PBS before cell-associated fluorescence was measured using an Accuri C6 Flow cytometer and analysed using Accuri C6 software (BD Biosciences).

#### Confocal microscopy

To confirm the uptake and internalization of STBEVs into HCAECs, live cell confocal laser scanning microscopy was performed following treatment with normal or PE STBEVs. Cells were grown in glass bottom chamber slides, and 10–20 μg/ml of PKH-labeled STBEVs added to subconfluent cells in phenol red-free and serum-free conditions and incubated for 1 hour. Surface-bound STBEVs were removed by extensive washing with 1 M NaCl and serum-free medium, followed by live cell imaging of intracellular STBEV in phenol-red free medium. The acquisition of images was performed using Zeiss LSM 710 confocal scanning microscope equipped with excitation laser wavelengths of 405, 488, and 633 nm, and a C-Apochromat 63X/1.20 W korr M27 water or Plan-Apochromat 63X/1.40 DIC M27 oil immersion objective. Images were acquired using Zen 2011 software (Carl Zeiss). For live imaging, cells were transferred to a humidified 5% CO2, 21% O2 atmosphere incubator integrated with the confocal scanning equipment. Images were collected during a time series of approximately 10 min with 10 s cycle time.

### STBEV transfer of miRNAs to primary human coronary artery endothelial cells

#### Cell treatment

The HCAECs were seeded into 6 well plates at a concentration of 0.2 × 10^6^ cells per well. After reaching 80% confluency, media was changed and normal (n = 5) or PE (n = 5) STBEVs added (20ug/mL). After 6 hours incubation, medium was removed and the cells washed with 1xPBS before addition of lysis buffer. The lysate was transferred to eppendorf tubes and frozen at −80 °C. As controls (n = 6), PBS or no vehicle/treatment were used.

#### RNA extraction and quality assessment

Total RNA was extracted using mirVana™ miRNA Isolation Kit (Applied Biosystems, Foster City, CA, USA) according to manufacturer’s instruction. RNA quality and miRNA content was assessed with an Agilent 2100 Bioanalyzer (Agilent Technologies, Palo Alto, USA) using the Nano Assay to evaluate RNA integrity number (RIN) value and total RNA quality, as well as the Small RNA Assay to investigate miRNA percentage and quality. A RIN value of 10 corresponds to intact RNA, and 1 to totally degraded RNA. The analysis confirmed the presence of high quality miRNA and RNA, which showed RIN values above 8.3 in all samples.

#### Profiling of placental miRNAs using a custom made miRNA array

To investigate the transfer of miRNAs from placental STBEVs, we designed a custom miRNA TaqMan® miRNA array card (Applied Biosystems). See Supplementary information Table S2, for a complete list of mature miRNA sequences from chromosome 19 miRNA cluster (C19MC). The cluster gives rise to 52 individual mature miRNAs accoding to the miRBase.org database. The 371–373 cluster gives rise to 8 individual mature miRNAs. Included on the array were also four well known miRNAs, which are widely expressed and relevant in placenta research; hsa-miR-141*, hsa-miR-210, hsa-miR-16^49^ and hsa-miR-222^50^.

Using the High Capacity cDNA Reverse Transcription Kit (Applied Biosystems), total RNA was transcribed to cDNA according to manufacturer’s instructions. The cDNA was mixed with TaqMan® Universal PCR Master Mix before addition to the array cards. The custom miRNA TaqMan® miRNA array cards were of format 64, which included 63 target assays (described above) and one endogenous control (U6 snRNA). Each card analysed two unique samples. qPCR was performed using standard protocol supplied by manufacturer for TaqMan® Array Micro Fluidic Cards on a QuantStudio 7 Flex (Applied Biosystems). Data were normalised to the endogenous control U6 snRNA. Using the Thermo Fisher Cloud software (Thermo Fisher Scientific, 81 Wyman Street Waltham, MA USA 02451), the Comparative C_T_ Method (ΔΔ C_T_ Method, Applied Biosystems) was used, according to manufacturer’s instructions, to calculate fold differences relative to samples treated with normal STBEVs.

#### Real time quantitative PCR for miRNA analysis

Following miRNA array analysis, four miRNAs were selected based on changes in expression; mir-517c, mir-517a, mir-519a and mir-210. These specific miRNAs were verified and analysed with RTqPCR in cells treated with normal or PE STBEVs.

Total RNA was extracted using the mirVana™ miRNA Isolation Kit, as described above. Ten ng RNA was transcribed using TaqMan® MicroRNA Reverse Transcription Kit (Applied Biosystems) according to manufacturer’s instructions. The following pre-designed TaqMan® MicroRNA assays (Applied Biosystems) were analysed: homo sapiens-microRNA-517c (hsa-mir-517c), hsa-mir-517a, hsa-mir-519a, hsa-mir-210 and U6 snRNA (sequences according to Supplementary information S2).

qPCR was performed using the standard protocol supplied by the manufacturer for TaqMan® MicroRNA Assays on an ABI PRISM 7000 sequence detection system (Applied Biosystems). Primers and probes were as described above. Each reaction was run in duplicate. Negative controls with no template as well as no reverse transcriptase controls were included for every miRNA primer pair. Data were normalized to U6 snRNA. Using the ΔΔC_T_ method described by Livak *et al*.^51^, fold differences were calculated relative to samples treated with normal STBEVs for the C19MC miRNAs, since these miRNAs were not detected in control samples. However, for mir-210, fold differences were calculated relative to control cells, i.e. untreated cells, for mir-210. A p-value < 0.05 was considered statistically significant. A fold change of >1.5 was defined as an increased miRNA content, while a fold change <1.5 was defined as a decrease.

### Predicted miRNA target genes

#### Rationale

Current understanding of miRNA binding to target genes suggest that the 5′ end of the miRNA, via the seed sequence of 7–8 nucleotides is important and is conserved between species. Positions 1–10 are important for binding to Agonaute (Ago) in the RNA induced silencing complex (RISC) and bases 2–6 interact with mRNA. Binding to Ago requires a helical confirmation involving energy considerations. Another consideration is how much ‘imperfection’ in base-pairing can be tolerated in miRNA-mRNA interactions given that miRNA can bind to multiple places on one mRNA, and many different miRNA can bind to one mRNA. The miRNA binding is considered to take place on the mRNA 3′ end but the coding region is also possible. Most computational prediction tools for miRNA target genes are considered to provide 30% precision^52–54^. All predicted targets need validation. Taking these considerations into account, the strategy used in this study was based on using predication models that focused on seed sequence stringent pairing, thermodynamic considerations, conserved seed sequences and where possible experimentally validated miRNA-mRNA interactions. Thus, the prediction programs chosen used multiple criteria. Unfortunately C19MC miRNA are primate-specific and therefore conservation criteria are limited, although TargetScan has *Pan troglodytes* data. Target genes, if retrieved by several different computational prediction tools, were selected for further analysis via literature searching for interactions in the placenta or cardiovascular function.

#### Prediction algorithms

Using the prediction algorithms DIANA-microT-CDS (diana.imis.athena-innovation.gr/DianaTools/index.php?r=microT_CDS/index)^55^, TargetScan (v7.0; targetscan.org)^56^ and miRmap (mirmap.ezlab.org)^57^, we identified potential miRNA target genes for the C19MC miRNAs mir-517a, mir-517c and mir-519a. By combining the gene lists from the three prediction algorithms, we found 33 genes for mir-517a and mir-517c as well as 872 genes for mir-519a. Using TarBase (v7.0; diana.imis.athena-innovation.gr/DianaTools/index.php?r=tarbase/index)^58^, experimentally supported miRNA targets were identified. Tarbase provided one (1) experimentally supported gene for mir-517a, no genes for mir-517c and four (4) genes for mir-519a. The literature was searched for possible gene functions. The following genes: FLT1, TGFBR2, PDGFD, ESR1, VLDLR and PDCD1LG2 (Fig. 5), were selected for further analysis based on their algorithm prediction scores as well as being known to be involved in placental and/or cardiovascular function as well as PE. The gene expression levels were examined using RTqPCR. All genes, except FLT1, were predicted target genes by all three prediction databases. FLT1 was chosen due to its significance in PE research as well as being a validated target for mir-517a/b and mir-517c^59^.

#### Real time quantitative PCR for target gene analysis

The HCAECs were, as described above, treated with normal or PE STBEVs. Total RNA was extracted, and transcribed using TaqMan® Reverse Transcription Reagents according to manufacturer’s instructions (Applied Biosystems). The following pre-designed qPCR assays (Applied Biosystems) were analysed: FLT1, TGFBR2, PDGFD, ESR1, VLDLR, PDCD1LG2, HMOX1, and GAPDH. RTqPCR was performed as described above.

### Transmission Electron Microscopy

#### STBEV characterisation

The STBEVs were visualized and identified using TEM, as previously described^60^. Briefly, TEM was performed using the NDOG2 antibody against PLAP (made in-house and provided by Prof Sargent), the microRNA Assay primer hsa-mir-517c (Applied Biosystems Inc., Foster City, CA, USA) and an antibody against the human foetal Hb (HbF) protein. All antibodies and primers were labelled with colloidal gold (BBI International) of different sizes; NDOG2 (anti-PLAP, 20 nm), mir-517c primer (5 nm) and anti-HbF antibody (5 nm). The samples were processed for negative staining and sections were examined with a transmission electron microscope (CM100 Twin, Philips, Eindhoven, Holland) operated at a 60 kV accelerating voltage. The images were recorded with a side-mounted Olympus Veleta camera (Olympus, Münster, Germany).

#### Cell incubation with foetal haemoglobin

To evaluate the effect of free HbF on STBEV uptake and miRNA transfer, HbF (0.7 mg/mL) was added to the cultured cells, with or without STBEVs. HbF was prepared from human cord blood and diluted in 15 mM Tris-HCl pH 8.0, 20 mM NaCl according to a previously published protocol^14^.

#### Cell analysis

For cell analysis, the HCAECs were incubated for 6 hours with normal or PE STBEVs, or normal STBEVs with HbF. Cells treated with 1xPBS only were used as controls. The TEM procedure was as described above. For a quantitative evaluation, 100 cellular profiles (n = 100) were analysed in each case from random distributions on different locations.

### Statistical analysis

Statistical analysis was performed using Origin 9 software (Microcal, Northampton, MA, USA). The non-parametric Mann-Whitney *U*-test was used and a p-value < 0.05 considered statistically significant.

## Electronic supplementary material

S1 Supplementary video

Supplementary information
