# Supplementary material for: Syncytiotrophoblast derived extracellular vesicles transfer functional placental miRNAs to primary human endothelial cells
Source: Sci Rep. 2017 Jul 4;7:4558. doi: 10.1038/s41598-017-04468-0 (PMC5496854; doi:10.1038/s41598-017-04468-0)
Supplement: Supplementary file 2 — Supplementary information [file 41598_2017_4468_MOESM2_ESM.pdf]

Supplementary information

Syncytiotrophoblast derived extracellular vesicles transfer functional placental miRNAs to primary human endothelial cells

Tina Cronqvist[1*], Dionne Tannetta[2], Matthias Mörgelin[3], Mattias Belting[4], Ian Sargent[2], Mary Familari[5,6], Stefan R. Hansson[1,6]

S1. Video, live cell confocal laser scanning microscopy

See separate video file named S2 Supplementary video - live cell confocal laser scanning microscopy.

S1. Figure legend

Using live cell confocal laser scanning microscopy, we could confirm the uptake of PKH-labelled STBEVs into the primary endothelial cells. The STBEVs appear to be localised near the nucleus at 60 min of internalization.

S2. Table mature miRNA sequences

Micro-RNA	Mature micro-RNA sequence	Assay ID
hsa-miR-520e	AAAGUGCUUCCUUUUUGAGGG	1119
hsa-miR-519d #	CAAAGUGCCUCCCUUUAGAGUG	2403
hsa-miR-518d-5p	CUCUAGAGGGAAGCACUUUCUG	2389
U6 snRNA #	GTGCTCGCTTCGGCAGCACATATACTAAAATTGGAACGATACAGAGAAGATTAGCATGGCCCCTGCGCAAGGATGACACGCAAATTCGTGAAGCGTTCCATATTTT	1973
hsa-miR-518e*	CUCUAGAGGGAAGCGCUUUCUG	2371
hsa-miR-520f	AAGUGCUUCCUUUUAGAGGGUU	1120
hsa-miR-516a-5p	UUCUCGAGGAAAGAAGCACUUUC	2416
hsa-miR-517*	CCUCUAGAUGGAAGCACUGUCU	1113
hsa-miR-518f*	CUCUAGAGGGAAGCACUUUCUC	2387
hsa-miR-519d-5p	CCUCCAAAGGGAAGCGCUUUCUGUU	466791_mat
hsa-miR-525-3p	GAAGGCGCUUCCCUUUAGAGCG	2385
hsa-miR-526b*	GAAAGUGCUUCCUUUUAGAGGC	2383
hsa-miR-520b	AAAGUGCUUCCUUUUAGAGGG	1116
hsa-miR-518c	CAAAGCGCUUCUCUUUAGAGUGU	2401
hsa-miR-522 #	AAAAUGGUUCCCUUUAGAGUGU	2413
hsa-miR-516b	AUCUGGAGGUAAGAAGCACUUU	1150
hsa-miR-1283	UCUACAAAGGAAAGCGCUUUCU	2890
hsa-miR-520g-5p	UCUAGAGGAAGCACUUUCUGUUU	466761_mat
hsa-miR-519e*	UUCUCCAAAAGGGAGCACUUUC	1166
hsa-miR-518a-3p	GAAAGCGCUUCCCUUUGCUGGA	2397
hsa-miR-519c	AAAGUGCAUCUUUUUAGAGGAU	1163
hsa-miR-526b	CUCUUGAGGGAAGCACUUUCUGU	2382
hsa-miR-520f-5p	CCUCUAAAGGGAAGCGCUUUCU	466371_mat
hsa-miR-519b-3p	AAAGUGCAUCCUUUUAGAGGUU	2384
hsa-miR-517c #	AUCGUGCAUCCUUUUAGAGUGU	1153
hsa-miR-520c-3p #	AAAGUGCUUCCUUUUAGAGGGU	2400
hsa-miR-518d	CAAAGCGCUUCCCUUUGGAGC	1159
hsa-miR-518a-5p	CUGCAAAGGGAAGCCCUUUC	2396

hsa-miR-520g #	ACAAAGUGCUUCCCUUUAGAGUGU	1121
hsa-miR-516-3p #	UGCUUCCUUUCAGAGGGU	1149
hsa-miR-520h	ACAAAGUGCUUCCCUUUAGAGU	1170
hsa-miR-517a #	AUCGUGCAUCCCUUUAGAGUGU	2402
hsa-miR-498	UUUCAAGCCAGGGGGCGUUUUUC	1044
hsa-miR-520d-5p #	CUACAAAGGGAAGCCCUUUC	2393
hsa-miR-512-3p #	AAGUGCUGUCAUAGCUGAGGUC	1823
hsa-miR-520a*	CUCCAGAGGGAAGUACUUUCU	1168
hsa-miR-519e	AAGUGCCUCCUUUUAGAGUGUU	2370
hsa-miR-515-5p	UUCUCCAAAAGAAAGCACUUUCUG	1112
hsa-miR-520a	AAAGUGCUUCCCUUUGGACUGU	1167
hsa-miR-519a #	AAAGUGCAUCCUUUUAGAGUGU	2415
hsa-miR-1323	UCAAAACUGAGGGGCAUUUUCU	2786
hsa-miR-520D-3P #	AAAGUGCUUCUCUUUGGUGGGU	2743
hsa-miR-523	GAACGCGCUUCCCUAUAGAGGGU	2386
hsa-miR-521	AACGCACUUCCCUUUAGAGUGU	1122
hsa-miR-515-3p	GAGUGCCUUCUUUUGGAGCGUU	2369
hsa-miR-518e #	AAAGCGCUUCCCUUCAGAGUG	2395
hsa-miR-518b	CAAAGCGCUCCCCUUUAGAGGU	1156
hsa-miR-518c*	UCUCUGGAGGGAAGCACUUUCUG	1158
hsa-miR-512-5p	CACUCAGCCUUGAGGGCACUUUC	1145
hsa-miR-525	CUCCAGAGGGAUGCACUUUCU	1174
hsa-miR-518f	GAAAGCGCUUCUCUUUAGAGG	2388
hsa-miR-524-5p	CUACAAAGGGAAGCACUUUCUC	1982
hsa-miR-524	GAAGGCGCUUCCCUUUGGAGU	1173
hsa-miR-372	AAAGUGCUGCGACAUUUGAGCGU	560
hsa-miR-371b-5p	ACUCAAAAGAUGGCGGCACUUU	463886_mat
hsa-miR-371b-3p	AAGUGCCCCCACAGUUUGAGUGC	464115_mat
hsa-miR-371-3p	AAGUGCCGCCAUCUUUUGAGUGU	2124
hsa-miR-373*	ACUCAAAAUGGGGGCGCUUUCC	562
hsa-miR-372-5p	CCUCAAAUGUGGAGCACUAUUCU	466501_mat
hsa-miR-373	GAAGUGCUUCGAUUUUGGGGUGU	561
hsa-miR-141*	CAUCUUCCAGUACAGUGUUGGA	2145

hsa-miR-210 #	CUGUGCGUGUGACAGCGGCUGA	512
hsa-miR-222 #	AGCUACAUCUGGCUACUGGGUCUC	525
hsa-miR-16 #	UAGCAGCACGUAAAUAUUGGCG	391

# = miRNA fulfilling the criteria of Ct<35 in one or more samples in the array analysis

S3. Heat map showing the analysis of miRNA array data, Ct values of miRNAs fulfilling expression inclusion criteria

	Control cells Ct	Normal STBEV Ct	PE STBEV Ct	
hsa-miR-16-000391	23.444	23.173	23.092	
hsa-miR-210-000512	31.172	30.713	30.845	p=0.022 comparing PE to normal
hsa-miR-222-000525	22.642	22.223	22.054	p=0.031 comparing control to normal
hsa-miR-516-3p-001149	33.207	33.496	33.492	
hsa-miR-520D-3P-002743	30.503	30.327	30.164	
hsa-miR-520d-5p-002393	32.062	31.831	31.488	
hsa-miR-512-3p-001823	-	33.949	34.25	
**hsa-miR-517a-002402**	**35.416**	**30.108**	**30.619**	**Selected for futher analysis by RTqPCR**
**hsa-miR-517c-001153**	**37.659**	**31.643**	**32.129**	**Selected for futher analysis by RTqPCR**
hsa-miR-518e-002395	-	33.339	33.688	
**hsa-miR-519a-002415**	**37.035**	**32.407**	**33.122**	**Selected for futher analysis by RTqPCR**
hsa-miR-519d-002403	-	33.068	32.545	
hsa-miR-520c-3p-002400	36.199	34.289	34.945	
hsa-miR-520g-001121	-	34.736	33.73	
hsa-miR-522-002413	36.186	34.404	33.869	

Lowest Ct values are marked red, highest Ct values are marked green. In white are values not fulfilling the criteria of Ct<35.

S4. Transmission electron microscopy figure characterising STBEVs

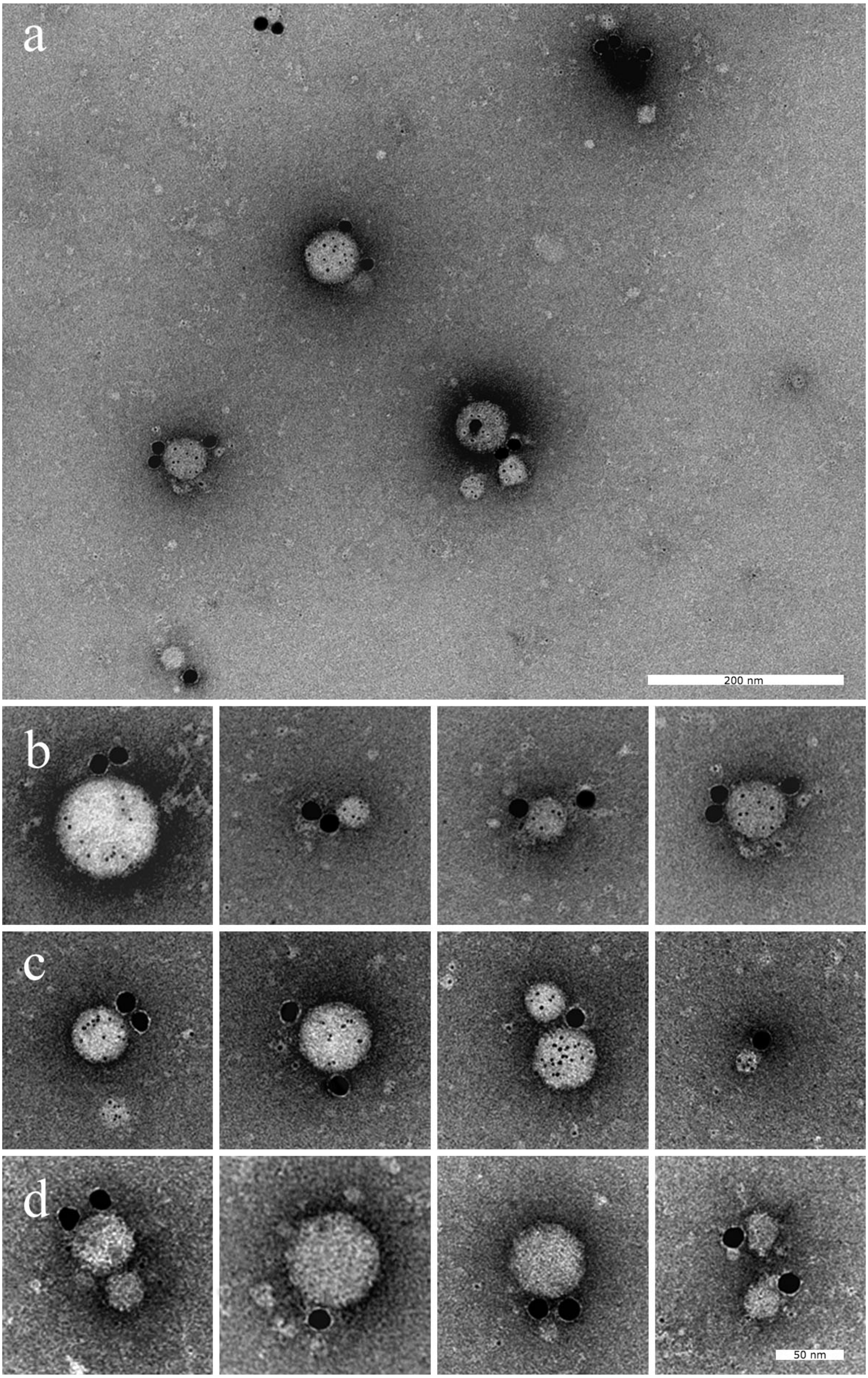

S4. Figure legend

STBEVs were visualized using transmission electron microscopy (TEM). Shown in panel (a) are PE STBEVs, treated with antibodies against PLAP and the miRNA primer against mir-517c, both labelled with colloidal gold of different sizes; 20 nm and 5 nm respectively. At higher magnification (b), PE STBEVs contained mir-517c in both PLAP positive and PLAP negative vesicles, as was also seen for normal STBEVs (data not shown). In (c) STBEVs were treated with antibodies against PLAP and HbF, labelled with colloidal gold of different sizes; 20 nm and 5 nm respectively. HbF was found inside both PLAP positive and PLAP negative PE vesicles (c). In contrast, normal STBEVs (d) were PLAP positive (20nm gold particles) but HbF negative (5nm gold particles).

S5. Full length western blots

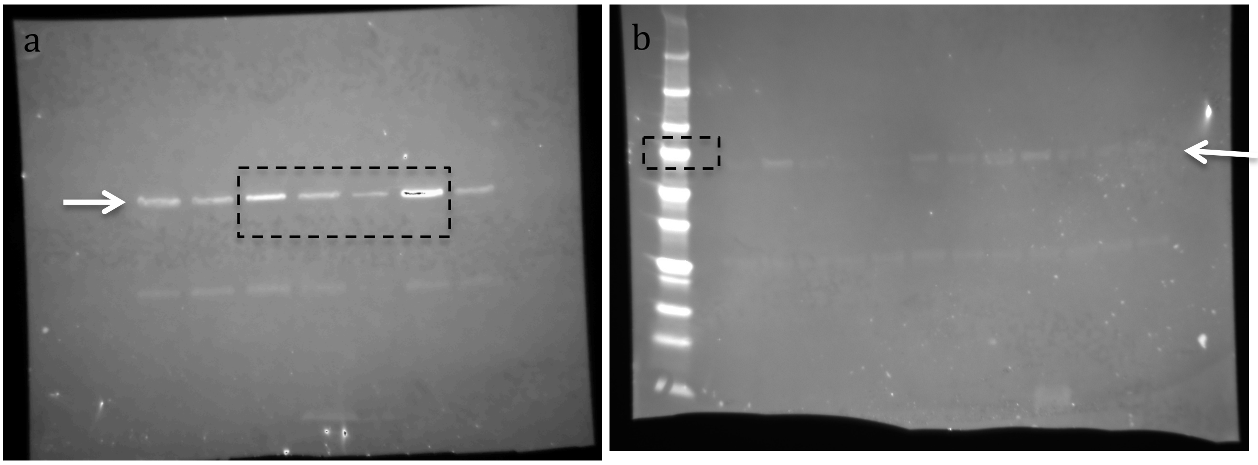

S5. Figure legend

Full length western blots for Figure 1. Characterisation of STBEVs using NTA and western blot. In S5a, lanes 1-4 represents normal STBEV samples, lanes 5-7 represents PE STBEV samples. In S5b, lanes 1-6 represent normal STBEV samples, lanes 7-12 represent PE STBEV samples. In both S5a and S5b, PLAP bands are indicated with an arrow. Marked are the sections shown in Figure 1, cropped and inverted for better visualisation – two normal and two PE STBEV samples (S5a) as well as the molecular weight 75kDa (S5b).
